# Cincinnati

**Published:** 1883-10

**Authors:** 


### CINCINNATI.

The city seems to be in a bad way. Her new Board of Health, according to the *Lancet and Clinic*, consists of five saloon-keepers and a quack doctor, the latter having, as one of his advertized specialties, a peculiar operation for the restoration of lost virginity. The doctor may be a thoroughly efficient sanitarian—we never heard of him before—but his operation so manifestly supplies a long-felt want in Cincinnati, that we fear he can hardly find time for the proper discharge of his official duties.—*Columbus Med. Journal.*

